# Editorial for the Special Issue on Heat and Mass Transfer in Micro/Nanosystems

**DOI:** 10.3390/mi13071151

**Published:** 2022-07-21

**Authors:** Ruijin Wang, Junfeng Zhang

**Affiliations:** 1 School of Mechanical Engineering, Hangzhou Dianzi University, Hangzhou 310018, China; 2 Bharti School of Engineering and Computer Science, Laurentian University, 935 Ramsey Lake Road, Sudbury, ON P3E 2C6, Canada

The miniaturization of components in mechanical and electronic equipment has been the driving force for the fast development of micro/nanosystems. Heat and mass transfer are crucial processes in such systems, and they have attracted great interest in recent years. Tremendous effort, in terms of theoretical analyses, experimental measurements, numerical simulation, and practical applications, has been devoted to improve our understanding of complex heat and mass transfer processes and behaviors in such micro/nanosystems.

This Special Issue is dedicated to showcase recent advances in heat and mass transfer in micro- and nanosystems, with particular focus on the development of new model and theory, the employment of new experimental techniques, the adoption of new computational methods, and the design of novel micro/nanodevices. Thirteen articles have been published after peer-review evaluations, and these articles cover a wide spectrum of active research in the frontiers of micro/nanosystems. For example, Hu et al. [1] studied the satellite droplet generation in piezoelectric methods, and found that there are two key parameters responsible for this phenomenon: the pulse frequency for driving the piezoelectric transducer tube and the fluid flow rate. Optimal operation conditions have also been proposed to eliminate the satellite droplets for deionized water. In the article by Song et al. [2], the authors developed a structural design to visualize the evaporation and condensation processes in the silicon-based ultra-thin loop heat pipe (s-UTLHP), and performed experimental measurements to study the heat transfer mechanism in such devices. To gain a more accurate thermal measurement for microfluidic devices, Meng et al. [3] proposed the use of a liquid metal to fill the gap space between the temperature sensor and the microfluidic substrate, and they also tested this concept on a microchennel chip with gallium. Furthermore, Wang et al. [4] developed a disposable microfluidic chip for the real-time monitoring of sweat rate. This economical and convenient paper-based sticker has a diameter of 25 mm and a thickness of 0.3 mm, and it can be applied to the skin at any parts of the body. The chip consists of multiple layers; in particular, the sweat-sensing layer has an impressed wax micro-channel containing chromogenic agent to show sweat absorption amount, which can be read directly from the scale lines on the chip surface. The proposed chip, as a low-cost and convenient wearable device, has potential applications in the real-time monitoring of sweat loss for bodybuilders, athletes, firefighters, etc.

With the rapid advances in computational technologies, numerical modeling and simulations have been proven as a valuable complement for studying complex systems and processes. In this direction, Saghir and Ranman [5] numerically investigated the thermal and hydraulic performances through minichannels with different pin-fin configurations, and their results showed that the wavy pin-fin configuration exhibited the best performance with a high Nusselt number and a low pressure drop. On the other hand, Jbeili and Zhang [6] examined the convective heat transfer performances of flows through porous materials, and found that, in addition to the porosity, the aspect ration of the microscopic porous structure and the possible interfacial thermal resistance can also affect the macroscopic thermal performance of the porous media. For an efficient evaluation of the flow and pressure distributions in a microchannel network, Zhao et al. [7] introduced an electric circuit analogy and applied it to study the effect of microchannel length on the flow behaviors. Another interesting study is presented by Huang et al. [8], where the immersed boundary method has been combined with the lattice Boltzmann method to study the trajectory of a neutrally buoyant circular particle in the pulsatile channel flow. The particle exhibits rich dynamic behaviors which have not been observed in non-pulsatile situations, and the results could be useful for nanoparticle transport in drug delivery applications.

Nanofluids, which are fluid suspensions of nanoparticles, have attracted great attention from scientists for the enhanced thermal performances, and extensive studies have been conducted over the past decades. In this Special Issue, several papers have been devoted to exploring the thermal enhancement mechanisms of nanofluids in various situations. Elsafy and Saghir [9] conducted simulations of the convective heat transfer by considering the nanofluids through straight and wavy microchannels filled with porous materials, and Wu and Zhang [10] studied the effects of the nanoparticle volume fraction of Al_2_O_3_-water nanofluids and the aspect ratio of rectangular microchannels on heat transfer and pumping power consumption for heat sink applications in electronic devices. Moreover, Rasool et al. [11,12] and Shafig et al. [13] established a mathematical framework to consider the magnetohydrodynamic flows of nanofluids in Darcy–Forchheimer porous media with moving boundaries.

The success of this Special Issue is built on the team work of everyone involved. We would like to thank the authors for contributing their interesting research, and we are also grateful to the anonymous reviewers for their critical comments which are valuable for further improving the article quality. Moreover, we should not forget to mention the help from Drs. Myung-Suk Chun, Khashayar Khoshmanesh, and Kwang-Yong Kim as Academic Editors, and Ms. Violet Cheng and Mr. Toot Jiang at the journal office. 
